# The Yin and Yang of IL-17 in Systemic Sclerosis

**DOI:** 10.3389/fimmu.2022.885609

**Published:** 2022-05-04

**Authors:** Lanxuan Wei, David Abraham, Voon Ong

**Affiliations:** Center for Rheumatology and Connective Tissue Disease, Department of Inflammation, Division of Medicine, University College London, London, United Kingdom

**Keywords:** IL-17, IL-17A, systemic sclerosis, fibrosis, inflammation

## Abstract

IL-17 (IL-17A) is a pro-inflammatory cytokine produced by a sub-set of T helper cells termed Th17 cells primarily in response to cytokines like TGF-β and IL-23 and play an important role in host defense. IL-17 signals *via* the IL-17RA/RC heterodimer and the adaptor protein Act1 to activate both canonical and non-canonical pathways inducing transcriptional activation and stabilization of mRNAs. IL-17 appears to act not directly on immune cells but stimulates stromal cells such as endothelial and epithelial cells and fibroblasts to secrete other immunomodulatory factors. Fibroblast activated by IL-17 can support the growth and differentiation of immune cells. Studies have begun to uncover a dual role for IL-17; on one hand enhancing immune reactions and promoting inflammatory diseases and on the other decreasing responses and immune activity in established disease settings. The balance of double-edged sword effect of IL-17 and autoimmunity is illustrated in a variety of human diseases and experimental models of diseases. Specifically, the emerging interest in autoimmunity in systemic sclerosis (Scleroderma, SSc) has led to potential role of IL-17A as a target therapy in this disease.

## Introduction: IL-17 and Th17 Cells

IL-17 family of cytokines comprises of different members which include IL-17B, IL-17C, IL-17D (also known as IL-27), IL-17E (also known as IL-25) and IL-17F (1), with IL-17A being the most studied one. IL-17 has pleiotropic functional impact on various cell types in human body, contributing to host defense against opportunistic pathogens infection (2) such as Candida and occurrence of chronic inflammatory disorders (2).

Most IL-17-secreting cells belong to the lymphoid lineage including natural killer (NK) cells, NK T cells (NKT), type 3 innate lymphoid cells (ILC3), gamma/delta T cells, and CD4+ T cells (1). Th17 cells are a group of CD4^+^ helper T cells primarily located in barrier organs in steady state (2) providing a defensive role upon the entry of pathogens. Their principal products are IL-17A, IL-17F, IL-21, and IL-22 (1). It was found that TGF-β, IL-21, IL-6, IL-1β are all critical mediators of the process of Th17 cell differentiation (3). TGF-β and IL-6 (4) can promote the recruitment and phosphorylation of STAT3 (5), leading to induction of the transcription factor retinoic acid receptor-related orphan nuclear receptor gamma t (RORγt) (2) which is the essential and specific regulator in Th17 cells (5). Regulatory T cells (Tregs) are a group of T cells that function to monitor and contain abnormal activation of immune system. Interleukin-1β and IL-6 downregulate while TGF-β induces transcription factor of Tregs (1). This indicates the delicate conversion between these two separate cell lines and the equilibrium between which (6) play a significant and positive part in the maintenance of immune homeostasis (2). Inhibition of Tregs and Th1 signals/enhancement of Th17 and Th2 signals (6) has been indicated to contribute to the pathogenesis of SSc. PGI_2_ analogues are also suggested to induce Th17 while inhibit Th1 cell responses in SSc (7). IL-23, a heterodimer consisting of an IL-12/IL-23 common p40 subunit and an IL-23 specific p19 subunit (3), stimulates various pathways to promote generation and stabilization of final mature Th17 cells (8). Blimp-1 has been suggested to be an essential transcription factor downstream of IL-23 signaling pathway that acts together with RORγt during the process of differentiation of Th17 cells (8).

**Figure 1:**
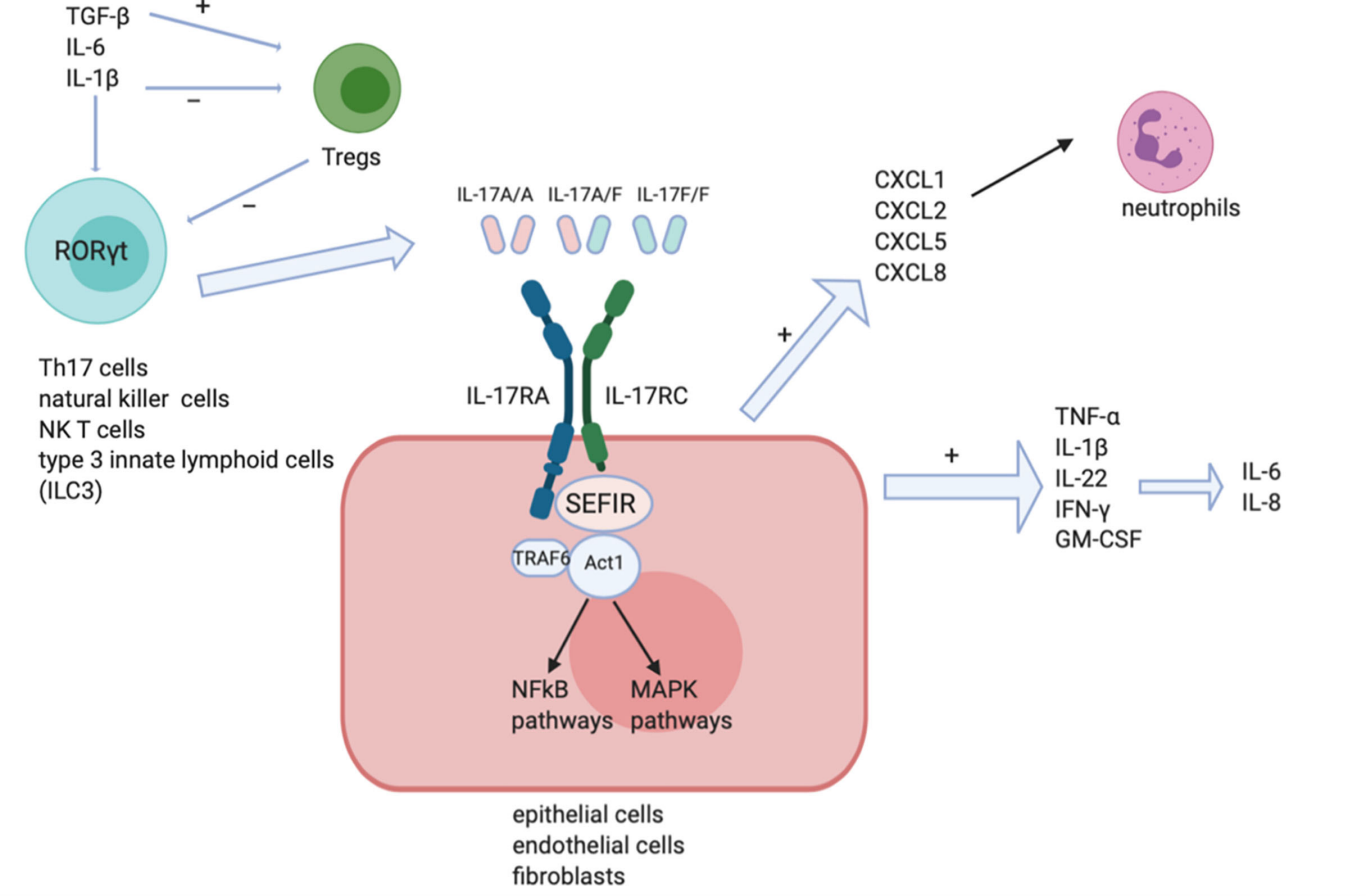
IL-17 signaling pathways. IL-17A/A homodimer, IL-17F/F homodimer and IL-17A/F heterodimer can each stimulate IL-17RA and IL-17RC receptor complexes on the surface of target cells like epithelial cells, endothelial cells and fibroblasts. The SEFIR motif recruits Act1 leading to the induction of downstream NF-kB and MAPK pathways. Pro-inflammatory cytokines like IL-1, IFN-γ, and IL-8 are then induced.

IL-17 receptor is found to express ubiquitously in multiple cell lines (8), resulting to the release of an extensive range of cytokines in epithelial cells, endothelial cells and fibroblasts (6) (as shown in **Figure 1**). The IL-17 receptor family is composed of five members (IL-17RA, IL-17RB, IL-17RC, IL-17RD, and IL-17RE) (9). All IL-17 receptor family contain a similar intracellular conserved cytoplasmic SEF/IL‐17R (SEFIR) motif (2). IL-17A is secreted either as homodimer of IL-17A/IL-17A or as IL-17A/IL-17F heterodimer (6), and they signal through the same receptor subunits comprised of an IL‐17RA chain and an IL‐17RC chain (1, 6). IL-17F among other IL-17 family members, is the most analogous one to IL-17A, sharing more than half of similarities structurally with IL-17A. Nevertheless IL-17F signaling strength is much weaker (6) than that of IL-17A possibly due to its weaker affinity upon binding with receptors (10). IL-17A binds with the IL- 17RA/RC receptor complex in order to exert its function. Act1, a ubiquitin ligase in its downstream pathway is recruited to further recruit tumor necrosis factor (TNF) receptor-associated factor 6 (TRAF6). TRAF6 is ubiquitylated by Act1 then activates the nuclear factor kappa B (NF-kB) and mitogen-activated protein (MAP) kinase pathways (2), including extracellular signal‐regulated kinase (ERK), p38 and JUN N‐terminal kinase (JNK) (11).

## Role of IL-17 in Inflammation

IL-17 exerts a protective role against many infectious diseases, while promoting inflammatory pathology in autoimmune diseases at the same time (8). The delicate control over the balance between protective and pathogenic sides of IL-17 is worthy of further investigation to achieve optimal regulation (12). IL-17 exerts its protective role in barrier immunity through promoting the production of antimicrobial factors and contributing to the recruitment of neutrophils (13). Defective IL-17 secretion could lead to chronic mucocutaneous candidiasis (14) thus confirming its protective function in mucosal immunity. IL‐17 exerts its pro-inflammatory roles by inducing various pro-inflammatory genes in its downstream signaling pathways. For instance, IL-17A could induce in target cells the expression of neutrophil chemoattractants such as CXCL1, CXCL2, CXCL5, and CXCL8; CCL20 is expressed stronger under IL-17A stimulation which promotes the recruitment of lymphocytes; growth factors such as G-CSF and GM-CSF are upregulated as well (2, 6, 11). In the meantime CCL20 also facilitates the recruitment of Th17 cells to the site where inflammation occurs thus creating an auto-enhancing loop of Th-17 related pathways (2). IL-17 is significant in its capacity to synergize with other cytokines such as TNF-α, IL-1β, IL-22, IFN-γ, and GM-CSF to enhance production of inflammatory mediators like IL-6 and IL-8 (2). Katz et al. reported in 2000 the regulatory effect of IL-17 in the process of complement protein synthesis. IL-17 alone could stimulate C3 synthesis while IL-17 and TNF-α synergize together to induce factor B production. Pro-inflammatory role of IL-17 is further proved considering the participation of C3 and factor B in immunobiological activities (15). C9 proteins are involved in local inflammatory responses in alveolar epithelial cells. Cipolla et al. first suggested in their studies that IL-17A actively induce C9 cascade in lung fibrosis. This participation in epithelial injury and fibrosis is achieved *via* p38MAPK signaling pathway activation. Antagonizing IL-17A to ameliorate C9 cascade could provide new strategies to abrogate lung fibrosis (16). In all, IL-17 is a key mediator in inflammatory reactions happening in the context of an environment under the influence of mutual actions of various different kinds of cytokines (12).

IL-17 and other Th17 cytokines can contribute to the pathogenesis of a diverse range of multiple autoimmune and inflammatory diseases (8), including psoriasis, inflammatory bowel disease (IBD), rheumatoid arthritis (RA), and multiple sclerosis (MS) (4). In rheumatoid arthritis, for example, IL-17-induced cytokines has a pro-inflammatory impact on flare-ups of this disease (8). IL-6 induced by IL-17 maintains the Th17-cell population, creating a self-reinforcing loop thus promoting and maintaining this chronic inflammatory state of RA (8). IL-17 family cytokines are also known to play a role in cancer immunity. IL-17A/F could manipulate immune cells such as macrophages leading to progression of lung cancer cell growth (17). IL-17-induced EMT contributes to the evolvement of lung cancer, actively stimulating cell migration and invasion *via* its downstream mediator NF-κB (18). The ‘‘IL-23-IL-17 axis’’ is also suggested to be a key driver and IL-23 and IL-17A are critical mediators of autoimmune diseases like psoriasis (19, 20).

## IL-17 and Systemic Sclerosis

Systemic Sclerosis (SSc) is a disease characterized by ECM deposition and diffuse fibrosis of the skin and internal organs (21). Its etiology remains a mystery yet to be discovered. Its etiology in particular autoimmunity has drawn significant interests recently and the emerging positive results from recent clinical trials targeting key immune pathways are testaments to the key role of autoimmunity in SSc pathogenesis (22). The interactions between various altered cell types such as epithelial cells, endothelial cells and immune cells and the pathogenic molecules they secreted resulted in typical changes in fibroblasts properties (21). T cells in particular are said to act an active part in this process (23), and play a prominent role in the pathogenesis of SSc. Being at the site of fibrosis, the soluble profibrotic mediators (IL-4, IL-6, IL-13) released by Th2 cells and their interactions with fibroblasts promoted the deposition of excess ECM and induced fibrosis in SSc (24).

Fibroblasts are a group of cells that can be originated from various different cell types. Once activated during the abnormal pathogenic process, they can transdifferentiate into myofibroblasts (25), which express α-smooth muscle cell actin (α-SMA), rendering its contractile capacity (26). Stimulated by mediators like IL-6, TGF-β, they can promote expression levels of type one collagen and fibronectin (26), contributing to the reprogramming of extracellular matrix and formation of pathological fibrosis of multiple organs. Key factors that are thought to play a critical role in the pathogenesis and progression of SSc are suggested as immunological disorder, environmental factors (silica exposure, chlorinated solvents, trichloroethylene, aromatic solvents), genetic factors and oxidative stress (21).

Abnormal T cell activation is a crucial part in the initiation and progression of systemic sclerosis (23). The subtle regulation between interplay of Th1/Th2 cells have long been the focus of stage. Either by releasing soluble mediators like IFN-γ (Th1 cells), IL-4 and IL-13(Th2 cells), or by contacting directly with fibroblasts, Th1 cells are thought to be anti-fibrotic by inhibiting ECM deposition and promoting MMP secretion (27), while Th2 cells are the opposite. Fibroblasts respond to the refined control over stimulation by Th1/Th2 cells and followed by secretion of mediators. These mediators are anti-angiogenic and anti-fibrotic in the context of Th1 cells induction, while others are pro-fibrotic and pro-angiogenic properties under Th2 cells stimulation (27). Fibroblasts act as ‘immune sentinels’ through a paracrine manner by releasing cytokines and having direct and indirect cell-cell interactions with immune cells. The ongoing bi-directional communication between immune cells and fibroblasts was considered the major driver of SSc. T cells contribute to endothelial dysfunction and the activation of macrophages and fibroblasts/myofibroblasts through cytokine while fibroblasts secrete ECM, collagens, glycosaminoglycans (GAGs) and fibronectin leading to fibrosis formation in SSc (28). Tregs exert anti-inflammatory role by releasing cytokines like IL-10 thus providing a protective role against aberrant immune activation. TGF-β is a critical inducer involved in the differentiation of Tregs but this induction is inhibited by IL-6 (29). Balance between Th17 cells and Tregs is regulated delicately in the development of SSc. Abnormal inflammatory changes occurred early in fibrosis, involving infiltration of mononuclear immune cells. Chizzolini et al. suggested that activated T cells are the dominant lymphocyte population in lesional skin and notably T cell infiltrates correlates with skin involvement suggesting an association between autoimmunity and fibrosis. Macrophages and mast cells have been suggested to participate in this course as well (27). The role of macrophages in progression of SSc is also worthy of inspection. Macrophages activated by Th1 cytokines produce pro-inflammatory cytokines, reducing ECM deposition, while macrophages activated by Th2 cytokines have an anti-inflammatory, profibrotic phenotype (27). These profibrotic macrophages are considered as activators in the fibrotic process. Though mostly related to vasculopathy, the function of complement in SSc pathogenesis is not fully known. C5a could induce CD4+ cells into a Th17 profile. Recent work highlighted that activated CD4+ T cells from early dcSSc expresses increased IL-17A and this is dependent on activation of intracellular inhibitory receptor, C5a receptor2. Biological coupling of perturbed intracellular complement (complosome) activation may be operational in an array of autoimmune rheumatic disease states and supports a unique role of Th1-driven pathology and complement activation. Notably, antagonizing C3a and C5a receptors leads to inhibition of EndoMT and abrogation of lung fibrosis in murine model (30, 31).

**Figure 2:**
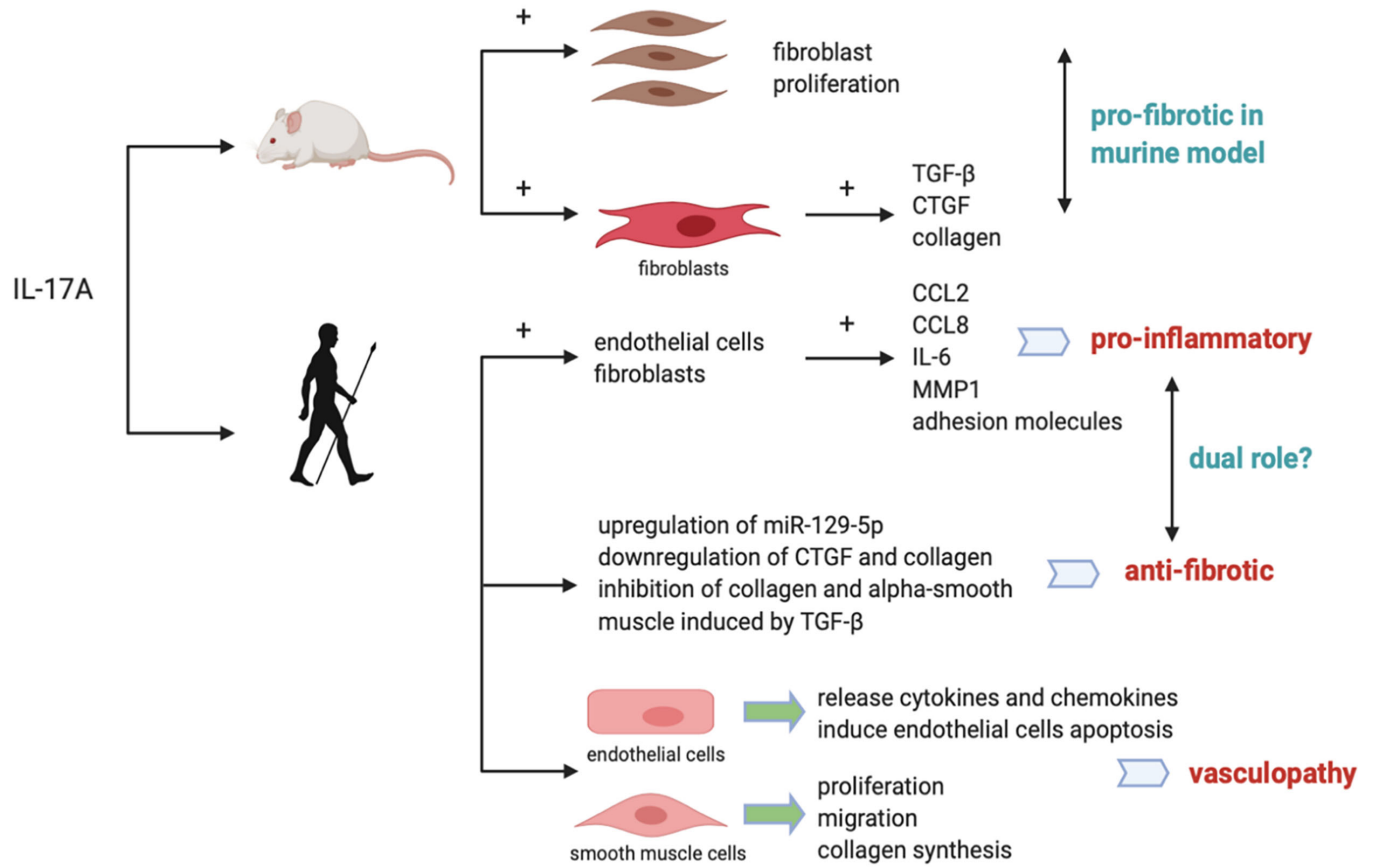
Proposed mechanisms for the role of IL-17A in SSc in mice and humans. In murine models, IL-17A is pro-fibrotic by promoting fibroblast proliferation and inducing synthesis of collagen and CTGF. In humans, IL-17A was reported to induce pro-inflammatory cytokines while inhibit collagen and CTGF synthesis, exerting a dual pro-inflammatory and anti-fibrotic role. IL-17A is also related with vasculopathy by promoting endothelial inflammation and smooth muscle cells proliferation, migration and collagen synthesis.

Previous studies have shown IL-17A plays a key role in the fibrotic process of various organs like lung, kidney, heart and skin. Both the levels of TGF-β and fibroblast TGF-β receptors are reported to be upregulated in the lungs of idiopathic pulmonary fibrosis patients (32). Except for TGF-β other cytokines involved in Th17 differentiation are indicated to induce lung fibrosis as well (32), suggesting potential role of IL-17A in pulmonary remodeling (33). By inhibiting Smad-independent pathways, IL-17A is thought to inhibit TGF-β-induced renal fibroblast activation (34). In animal models of skin fibrosis IL-17A is suggested to be a profibrotic mediator *via* TGF-β-dependent pathways to induce collagen deposition in skin (35). Systemic Sclerosis, characterized by fibrosis of multiple organs, is suggested to be related to a polarization to Th17 pathway-induced activation of immune responses (36) (more detailed proposed mechanisms shown in **Figure 2**).

Studies have shown that Th17 cell–derived IL-17 was significantly higher in the serum of SSc patients (36). Majority studies indicate that the levels of IL-17A in SSc serum is increased while contradictory studies indicating no differences or lower levels of IL-17A in SSc exist (1). Th17 response is regulated towards IL-17A pathway instead IL-17F (10). IL-17A+ cells are increased in the dermis of SSc skin (1) while IL-17F + cells were relatively low in Ssc skin (10). Circulating IL-17A in serum of SSc patients are high while IL-17F levels were not. Also IL-17A mRNA from Ssc lesional skin was higher while the trace of IL-17F mRNA was tiny (10). The numbers of IL-17+ cells are inversely proportional to the extent of severity in SSc (37). It has been demonstrated that IL-17 promoted the proliferation of SSc fibroblasts, but the effect on collagen and ECM protein synthesis is trivial (36). High IL-17E and low IL-17C has been reported in morphea as well (38). IL-21/IL-21R could be potential biomarkers presented in early SSc skin lesions indicating SSc is possibly a Th17-/Th22-driven disorder (39).

IL-17A is indicated to have profibrotic role in mouse models of lung fibrosis (35). As NF-κB signaling pathway is downstream of IL-17A and Act1 being the regulator of it, interference of Act1 leads to inhibition of pulmonary fibroblasts proliferation (13). Previous murine *in vivo* experiments show that IL-17A induces mouse skin fibroblasts to release TGF-β, CTGF and collagen; IL-17 can also promote ECM deposition and epithelial-to-mesenchymal transition in mouse alveolar epithelial cells *via* TGF-β-dependent pathways (23). We can see that IL-17A does have a pro-fibrotic activity in animal models. Karatas et al. reported that secukinumab and metformin ameliorated dermal fibrosis in bleomycin-induced mouse models by decreasing dermal thickness and tissue IL-17A levels, suggesting the association of IL-17A with dermal fibrosis in animals (40). However the pro-fibrotic mechanisms of IL-17A in animal models cannot be applied to humans (1). No definitive evidence can prove the role of IL-17A in lung fibrosis progression involved in SSc patients (1).The dichotomous nature of IL-17 lies in that it is pro-fibrotic in animal models while having a dual pro-inflammatory and anti-fibrotic role in humans (36). This dual role of IL-17A in SSc patients may make it capable of inducing the inflammatory responses while protecting against fibrosis at the same time (41). Zhou et al. have found that IL-17RA is expressed mainly in epidermis in SSc, while keratinocyte-derived mediators are capable of crossing the epidermal–dermal basement membrane. Fibroblasts, endothelial cells, and leukocytes located in dermis are thus stimulated (41). Dufour et al. observe an anti-fibrotic activity of IL-17A (42) in organotypic full-skin cultures in the context of simulated physiological condition under the synergistic influence of many cell types and cytokines together (1), further confirming the role of IL-17A in promoting pro-inflammatory and anti-fibrotic responses (43). Vettori et al. found that IL-17A regulates T cell-mediated antifibrotic and proapoptotic role in skin fibroblasts (44). The pro-inflammatory property of IL-17A is that it promotes production of CCL2, CCL8, IL-6, MMP1, and the expression of adhesion molecules in dermal fibroblasts and endothelial cells (1). Park et al. reported in two different murine models of SSc, IL-1β and IL-17A showed synergistic effects on inducing pro-inflammatory mediators like IL-6, MMP-9 and TGF-β (45). Thus, targeting IL-1 and IL-17A activity could provide insights into novel treatment strategy in SSc (45). The anti-fibrogenic property of IL-17A is that it inhibits collagen production or alpha-smooth muscle actin expression induced by TGF-β in dermal fibroblasts (1). Dufour et al. reported first the dual role of IL-17A under the influence of TGF-β. IL-17A could exert pro-fibrotic function by synergizing with TGF-β to induce more robust IL-6 production, and in the meantime exert anti-fibrotic function by inhibiting TGF-β-induced collagen synthesis. P38 MAPK signaling pathway is suggested to be the unique joint downstream pathway of this IL-17A and TGF-β synergy (46). IL-17A can also exerts anti-fibrogenic effect *via* the upregulation of miR-129-5p and the downregulation of connective tissue growth factor (CTGF) and collagen. It has been suggested that intrinsic activation of TGF-β in SSc fibroblasts could in turn inhibit expression of IL-17 receptor in fibroblasts, leading to an amplification of collagen levels and fibrosis in SSc (47). One possible reason this anti-fibrogenic effect of IL-17A is not capable of containing the fibrosis in SSc could be that intrinsic differences between normal and SSc fibroblasts show that SSc fibroblasts are more resistant than their normal counterparts in response to collagen inhibition under the influence of IL-17. This property of SSc fibroblasts may help them escape or limit the anti-fibrotic effects of IL-17 (40).

As the earliest stage of the course of SSc, vasculopathy occurred before fibrosis in SSc skin (31). Release of proinflammatory mediator and vasoactive regulators mark the beginning of endothelial aberrant activation (48). Newly expressed adhesion molecules on the surface of endothelial cells facilitate inflammatory cells infiltration and interaction. Xing et al. discovered that IL-17A derived from sera of SSc patients mediates endothelial cell inflammation *via* ERK1/2 phosphorylation (48). IL-17A induces endothelial cells to release cytokines and chemokines that attract neutrophils infiltration. Also, IL-17 is said to induce endothelial cells apoptosis which further promote endothelial cell dysfunction in the context of abnormal inflammation (35).

IL-17A has pleiotropic effect on vascular smooth muscle cells from SSc patients. IL-17A promotes proliferation and migration of these cells, resemblance to the formation of atherosclerosis plug (10). Liu et al. discovered that IL-17A from SSc patients could induce the proliferation, migration and collagen synthesis of DVSMCs *via* ERK1/2 signaling pathway (49), IL-17A exerts its pro-fibrotic role in endothelial inflammation in a dose- and time-dependent pattern (49). IL-17A enhances release of adhesion molecules such as CCL-20, ICAM-1, CXCR-4 and VCAM-1 (10), attracting neutrophils migrating to the inflammation site. Xing et al. also found that T cell adherence to HUVECs is enhance by IL-17A (48).Whether targeting ERK1/2 might be a potential strategy for the treatment of SSc vasculopathy could be an interesting topic. Apart from IL-17A, Fukayama et al. found elevated levels of IL-17F and IL-17E is correlated with vasculopathy in SSc patients as well by interacting with receptors on vascular endothelial cells to induce endothelial cell proliferation (50).

## IL-17 Targeting Therapy

There are many types of antibodies that block a diverse range of area in IL-17 pathway in clinical use (as shown in **Table 1**). The first clinically used drug that antagonize IL-17A is Secukinumab approved in 2015 (51). Secukinumab and Ixekizumab inhibit IL-17A and IL-17A/F and can be used in the treatment of autoimmune diseases like psoriasis. Bimekizumab is developed to target both IL-17A and IL-17F at the site of the common motif they shared. Brodalumab antagonizes with the ubiquitously expressed common IL-17RA receptor subunit thus have the potential to inhibit different IL-17 family members at the same time, such as IL-17A, IL-17F, IL-17C and IL-17E (2, 19). It has been suggested that blocking IL-17A can also inhibit Calcium cascade which help to attenuate lung fibrosis (52). IL-23 is known to be the key mediator in the maturation of IL-17 and it is consisted of the IL-23-specific p19 subunit and the common p40 subunit (2). Antibodies against IL-23-specific p19 subunit include Tildrakizumab and Risankizumab (2, 51). Antibodies against common p40 subunit shared with IL-12 include Ustekinumab (51). As IL-17A synergizes with TNF-α, antibodies like ABT-122 could potentially target TNF-α at one site and IL-17A at the other (2). IL-17A inhibitors demonstrate a favorable safety, efficacy, and tolerability profile, bringing the success of treating of moderate-to-severe plaque psoriasis (53). Targeting STAT3, the upstream regulator of IL-17 differentiation has the potential to modulate fibrosis since many other signaling pathways in fibrotic events converge on STAT3 (49). However it is exactly because its central role in many immune processes that its off-target effect when targeting STAT3 needs further consideration (30). Since IL-17A signaling pathway involves activation of NF-ĸB, and Act1 is the upstream regulator of this pathway, silencing Act may provide new insights into next generation of therapies (13). Dual role of IL-17A and the subtle balance over its control raises concerns over the use of antibodies against IL-17——whether it will abrogate fibrosis or antagonize the protective role of IL-17 remains an issue and requires further investigations into it. It is worthy of consideration the protective role of IL-17A in host defense against infection when blocking IL-17 pathway, as it could bring detrimental harm (54).

**Table 1:** Summary of antibodies that target IL-17 pathways.

IL-17A and IL-17A/F inhibitors	Secukinumab
	Ixekizumab
IL-17A and IL-17F inhibitors	Bimekizumab
IL-17RA inhibitors	Brodalumab
IL-23 inhibitors	Tildrakizumab
	Risankizumab
	Ustekinumab
IL-17A and TNF-α inhibitors	ABT-122

## Concluding Remarks and Future

IL-17 (IL-17A) is a pro-inflammatory cytokine produced by a sub-set of T helper cells termed Th17 cells primarily in response to cytokines like TGF-β and IL-23. IL-17 plays an important role in host defense and is linked to various autoimmune diseases pathogenesis. IL-17 signals *via* the IL-17RA/RC heterodimer and the adaptor protein Act1 to activate NF-kB and MAPK pathways. IL-17 stimulates stromal cells such as endothelial, epithelial cells and fibroblasts to secrete other immunomodulatory factors. The pathogenic role of IL-17A in SSc is quite intriguing: on one hand IL-17A promotes secretion of pro-inflammatory cytokines and enhances immune reactions and on the other it decreases fibrotic responses and abrogates fibrosis. The potential target therapy against IL-17A pathways could provide new insights into treatment strategies of SSc and is worthy of further deeper investigation.

## Author Contributions

DA and VO contributed to helping draft the framework and giving suggestions of modifications for this review. LW as the first author of this review completed most of the writing. All authors contributed to the article and approved the submitted version.

## Conflict of Interest

The authors declare that the research was conducted in the absence of any commercial or financial relationships that could be construed as a potential conflict of interest.

## Publisher’s Note

All claims expressed in this article are solely those of the authors and do not necessarily represent those of their affiliated organizations, or those of the publisher, the editors and the reviewers. Any product that may be evaluated in this article, or claim that may be made by its manufacturer, is not guaranteed or endorsed by the publisher.
